# Predicting Therapeutic Efficacy of Transarterial Chemoembolization with Drug-Eluting Beads for Hepatocellular Carcinoma Using Contrast-Enhanced Ultrasound

**DOI:** 10.3390/diagnostics11020291

**Published:** 2021-02-12

**Authors:** Kazue Shiozawa, Takashi Matsui, Takahiro Murakami, Manabu Watanabe, Iruru Maetani

**Affiliations:** Division of Gastroenterology and Hepatology, Department of Internal Medicine, Toho University Ohashi Medical Center 2-22-36, Ohashi, Meguro-ku, Tokyo 153-8515, Japan; takashi.matsui@med.toho-u.ac.jp (T.M.); takahiro.murakami@med.toho-u.ac.jp (T.M.); manabu62@med.toho-u.ac.jp (M.W.); mtnir50637@med.toho-u.ac.jp (I.M.)

**Keywords:** hepatocellular carcinoma, contrast-enhanced ultrasound, transarterial chemoembolization with drug-eluting beads, intra-arterial contrast-enhanced ultrasound

## Abstract

The aim of this study was to assess the usefulness of contrast-enhanced ultrasound (CEUS) for predicting the therapeutic efficacy of transarterial chemoembolization with drug-eluting beads (DEB-TACE) for hepatocellular carcinoma (HCC). Thirty-two patients with HCC who underwent DEB-TACE were enrolled in this study. Enhancement patterns of vascular phase images on CEUS were compared before and within 3 days after DEB-TACE, and the patterns after DEB-TACE were classified as follows: Pattern A, no enhancement; Pattern B, peripheral ring enhancement; Pattern C, partial enhancement within or peripheral to tumors, and Pattern D, reduced or unchanged enhancement in the whole tumor. Enhancement patterns in all lesions and contrast-enhanced computed tomography (CECT) findings after DEB-TACE were compared statistically. The treatment response of DEB-TACE was evaluated using the Modified Response Evaluation Criteria in Solid Tumors (mRECIST) by CECT. The enhancement patterns on CEUS performed within 3 days after DEB-TACE were defined as Pattern A in 17 cases, B in 7, C in 13, and D in 2. The complete response rates at one month after treatment were 94.1% (16/17 lesions) for Pattern A, 85.7% (6/7) for B, 15.4% (2/13) for C, and 50% (1/2) for D. The response rates were significantly higher for lesions with Pattern A compared to those with Pattern C at one month (*p* = 0.009) and 12 months (*p* < 0.001) after treatment, and significantly higher for lesions with Pattern B compared to those with Pattern C at 12 months after treatment (*p* = 0.031). Comparisons between other patterns showed no significant differences. CEUS immediately after DEB-TACE may allow early assessment of therapeutic efficacy, with findings of no enhancement or peripheral ring enhancement suggesting a positive outcome.

## 1. Introduction

Contrast-enhanced ultrasound (CEUS) is a technique that enables tumor visualization without the use of ionizing radiation or the risk of nephrotoxicity associated with contrast-enhanced computed tomography (CECT) [1]. CEUS is useful for the assessment of the hemodynamics of hepatic tumors and surrounding hepatic parenchyma in real time. We evaluated the therapeutic efficacy of sorafenib (Nexavar^®^; Bayer Healthcare, Leverkusen, Germany) and CyberKnife^®^ (Accuray Incorporated, Sunnyvale, CA, USA) for hepatocellular carcinoma (HCC) using CEUS with Sonazoid^®^ (Daiichi Sankyo, Tokyo, Japan) and investigated the usefulness of CEUS [2,3].

Drug-eluting beads (DEBs) are polyvinyl alcohol-based microspheres that can be loaded with anthracycline drugs, such as doxorubicin [4]. Transarterial chemoembolization (TACE) with DEB (DEB-TACE) is now used in catheter-based locoregional therapy, which takes advantage of the arterial supply to HCC and spares the surrounding hepatic parenchyma, which receives most of its blood supply from the portal vein [5,6]. When injected through a catheter or microcatheter at the tumor site, DEBs act as embolic material, causing tumor ischemia, but they also release drugs in a sustained and controlled manner [7].

There have been several studies showing the usefulness of CECT and magnetic resonance imaging (MRI) for evaluation of the efficacy of DEB-TACE in patients with HCC [8,9], and Chung et al. [10] suggested that the efficacy of DEB-TACE is related to enhancement patterns on CECT. On the other hand, although there was a previous report evaluating the residual blood flow after DEB-TACE for HCC using CEUS [11], there has been no study that has verified if the early predicting therapeutic efficacy of DEB-TACE is related to enhancement patterns on CEUS. In the present study, we examined enhancement patterns on CEUS performed soon after DEB-TACE as potential predictors of therapeutic efficacy of DEB-TACE in patients with HCC.

## 2. Materials and Methods

### 2.1. Patient Characteristics

The subjects were 32 patients with HCC (39 lesions) who were treated with DEB-TACE from June 2015 to December 2017, and in whom all lesions were visualized by ultrasound (US). CEUS was performed within 3 days after DEB-TACE, and therapeutic evaluations with CECT were performed 1 and 3 months after DEB-TACE and every 3 months thereafter. The patients included 28 males and 4 females; the median age was 73 years old; the underlying liver disease was hepatitis B in 4 patients, hepatitis C in 12 patients, alcoholic liver disease in 14 patients, and non-alcoholic steatohepatitis in 2 patients; and the median tumor diameter was 21 mm. All patients were diagnosed with HCC using gray-scale US, CECT, and Gd-EOB-DTPA-enhanced MRI, based on the new guidelines of the American Association for the Study of Liver Disease [12]. Serum α-fetoprotein (AFP), AFP-L3 fraction, and des-γ-carboxyprothrombin (DCP) levels were used for diagnosis, as needed (Table 1).

We chose all patients based on the Evidence-based Clinical Practice Guidelines for HCC developed by the Japan Society of Hepatology [13]. All 32 patients had hepatic cirrhosis with Child–Pugh classification A or B and an Eastern Cooperative Oncology Group performance status < 2 [14]. All lesions were single HCC ≤ 50 mm in diameter or up to three HCCs ≤ 30 mm in diameter. Tumors were selected that were difficult to treat with radiofrequency ablation, such as those with the presence of ascites, close to vessels, or on the liver surface in patients who did not want surgical resection. Exclusion criteria were advanced-stage HCC in the BCLC classification, serum total bilirubin > 3 mg/dL, history of heart or renal impairment, iodine allergy, and egg allergy.

The study was performed in accordance with the Declaration of Helsinki, the International Conference on Harmonization Guidelines for Good Clinical Practice, and local laws and regulations. Approval was obtained from the Ethics Committee of Toho University Ohashi Medical Center. Informed consent for this study was obtained from all patients.

### 2.2. CEUS Imaging

Gray-scale US and CEUS using Sonazoid^®^ were performed within one month before DEB-TACE and within 3 days after DEB-TACE in all patients. All CEUS procedures were performed by two sonographers with 25 and 18 years of experience using an Aplio XG (Canon medical systems, Tokyo, Japan) with convex probe (PVT-375BT, 3.75-MHz center frequency). The mechanical index for the acoustic output was set to 0.2 and the dynamic range was set to 60–65 dB. In patients in whom lesions were detected by gray-scale US, the single focus point was set at the lower margin of the lesion. An intravenous bolus injection of Sonazoid^®^ (0.5 mL) was administered via a left cubital venous line, followed by flushing with 10 mL of normal saline. The dynamics of the enhancement of the lesion were observed in the vascular phase 0–60 s after Sonazoid^®^ injection, and in the post-vascular phase 10 min after Sonazoid^®^ injection. Subsequently, the feeding blood vessel was identified by re-injection of Sonazoid^®^ [15] in as many lesions as possible. Video images of all CEUS procedures were stored on the hard disk of the scanner and transferred to a high-performance personal computer.

### 2.3. CECT Imaging

All CECT examinations were performed using a 64 multidetector CT scanner (Light Speed V CT, GE Healthcare, Tokyo, Japan), and cephalocaudal images were obtained with section thicknesses of 3 mm and pitch 1.3, with intravenous bolus injection of non-ionic contrast material (90 mL of 300 mgI/dL; Iopamiron^®^, Bayer Schering Pharma, Osaka, Japan) at 3 mL/s via an antecubital vein. The scanning delay set for arterial phase and equilibrium phase was 30–40 s and 120–150 s, respectively.

The portal venous phase on CECT could not be obtained in 2/39 lesions (5.1%) in this study due to imaging errors. Therefore, the standard CT protocol not including the portal venous phase is described for all lesions.

### 2.4. DEB-TACE Procedure

CECT was performed within one month before DEB-TACE in all patients, a 3D-CT angiogram was prepared, and abdominal angiography was performed with reference to the CT findings. DEB-TACE was performed by an expert interventional radiologist and hepatologist with 31 years of experience and a hepatologist with 19 years of experience. The right groin and upper abdomen were cleansed with iodine, and the patient was draped under sterile cloths with exposure of the right groin and upper abdomen. Through a right femoral artery access and after placement of a 3-Fr shepherd hook catheter (Terumo, Tokyo, Japan), the celiac trunk was examined through the arterial and venous phases to define the hepatic artery anatomy and to assess portal vein patency. Then, the hepatic artery was selectively catheterized (segment/subsegment) to study the arterial supply of the target lesions. A coaxial microcatheter (Haruka^®^ (JMS Co., Ltd. Tokyo, Japan) or Wonder III^®^ (UTM Co., Ltd. Aichi, Japan)) was advanced in every feeding artery to allow embolization of the lesion with drug-eluting microspheres (DC beads^®^ (Eisai Co., Ltd. Tokyo, Japan), 100–300 μm in diameter, 1 vial) loaded with 50 mg of epirubicin^®^ (Nippon Kayaku Co., Ltd. Tokyo, Japan) until contrast medium disappeared from the blood vessel within 5–6 heart beats.

All treatments were performed with DEB-TACE using intra-arterial CEUS (IAUS). At this time, digital subtraction angiography and IAUS were performed and tumor enhancement was evaluated. If residual tumor enhancement was observed in any images, DEB-TACE was repeated and the disappearance of as much tumor enhancement as possible was confirmed using IAUS, after which the treatment was considered complete. IAUS was performed by administration of Sonazoid^®^ through the microcatheter and imaging of the area of the target lesion with a dedicated, contrast-specific technique. All IAUS procedures were performed by the same expert interventional radiologist and hepatologist with 31 years of experience using an Aplio 400 (Cannon Medical Systems, Tokyo, Japan) with a convex probe (PVT-375BT, 3.75-MHz center frequency). The MI for the acoustic output was 0.2–0.3 and the dynamic range was 60–65 dB. A single focal point was set at the deep site of the lesion. Sonazoid^®^ (0.5 mL diluted with 49.5 mL of distilled water) was used as the contrast medium in IAUS. The diluted Sonazoid^®^ was introduced into the feeding artery by intermittent injection of 0.3–0.5 mL through a microcatheter placed in the artery and flushing with saline at the same flow rate [16].

### 2.5. Image Analysis

**Figure 1:**
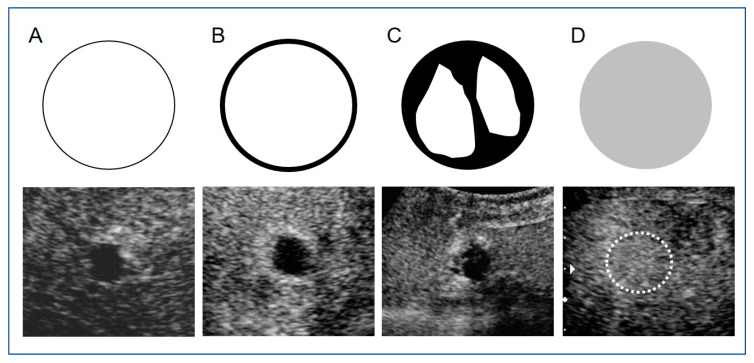
Diagrams and images of vascular enhancement patterns on contrast-enhanced ultrasound (CEUS) using Sonazoid^®^ within 3 days after transarterial chemoembolization with drug-eluting beads (DEB-TACE) for hepatocellular carcinoma (HCC). Upper images are diagrams and lower images are actual CEUS images of cases in this study. **A**: No enhancement; **B**: Peripheral ring enhancement; **C**: Partial enhancement within or peripheral tumors; **D**: Reduced or unchanged enhancements in whole tumors. (This CEUS image is a reduced enhancement in whole tumor (in white dot circle).

Two hepatologists (certified ultrasound specialists and instructors in the Japan Society of Ultrasonics in Medicine) with 26 and 11 years of experience reviewed the CEUS images collected within 3 days after DEB-TACE using IAUS. Image analyses were conducted in separate rooms to ensure independence in the findings. Lesions that were assessed differently by the two reviewers were subsequently discussed to reach a conclusion. Enhancement patterns of vascular phase images on CEUS for all lesions were compared before DEB-TACE and within 3 days after DEB-TACE, and the patterns within 3 days after DEB-TACE were classified as follows: Pattern A, no enhancement; Pattern B, peripheral ring enhancement; Pattern C, partial enhancement within or peripheral to tumors, and Pattern D, reduced or unchanged enhancement in the whole tumor (Figure 1). Complete peripheral ring enhancement included cases with a thin ring width and a location inside the treated tumor margin. Enhancements within or peripheral to tumors were defined as partial internal enhancement or peripheral nodule-like enhancement. Reduced or unchanged enhancement in the whole tumor was defined in comparison with the enhancement level before DEB-TACE. Enhancement patterns in all lesions and CECT findings one month after DEB-TACE and every 2–3 months thereafter were compared. The treatment response of DEB-TACE was evaluated using the Modified Response Evaluation Criteria in Solid Tumors (mRECIST: CR: complete response, PR: partial response, SD: stable disease, PD: progressive disease) by CECT [17]. TACE was additionally performed in all cases judged as PD on follow-up imaging after treatment. All lesions treated with additional TACE were considered to be PD in evaluating the therapeutic efficacy at 12 months after treatment.

### 2.6. Statistical Analysis

Local recurrence control rates were evaluated in all 39 lesions. Cases assessed as PD by CECT during follow-up were defined as having recurrence. Local recurrence control rates were determined by the Kaplan–Meier method, setting the baseline at the day of DEB-TACE. The mRECIST results assessed 1 and 12 months after treatment were classified as CR + PR (response) and SD + PD (non-response), and relationships with three enhancement patterns (excluding Pattern D) were assessed by Chi-squared test or Fisher exact test.

Further, the patient and tumor characteristics (tumor size, serum AFP and DCP levels) in each pattern were compared using the Kruskal–Wallis test. The level of significance of all analyses was *p* < 0.05.

The degree of inter-reviewer agreement for enhancement patterns of vascular phase images on CEUS was calculated with a kappa statistic. In general, a kappa value greater than 0.75 is considered excellent agreement; 0.4–0.75 is considered good agreement, and less than 0.4 is considered poor agreement.

## 3. Results

**Figure 2:**
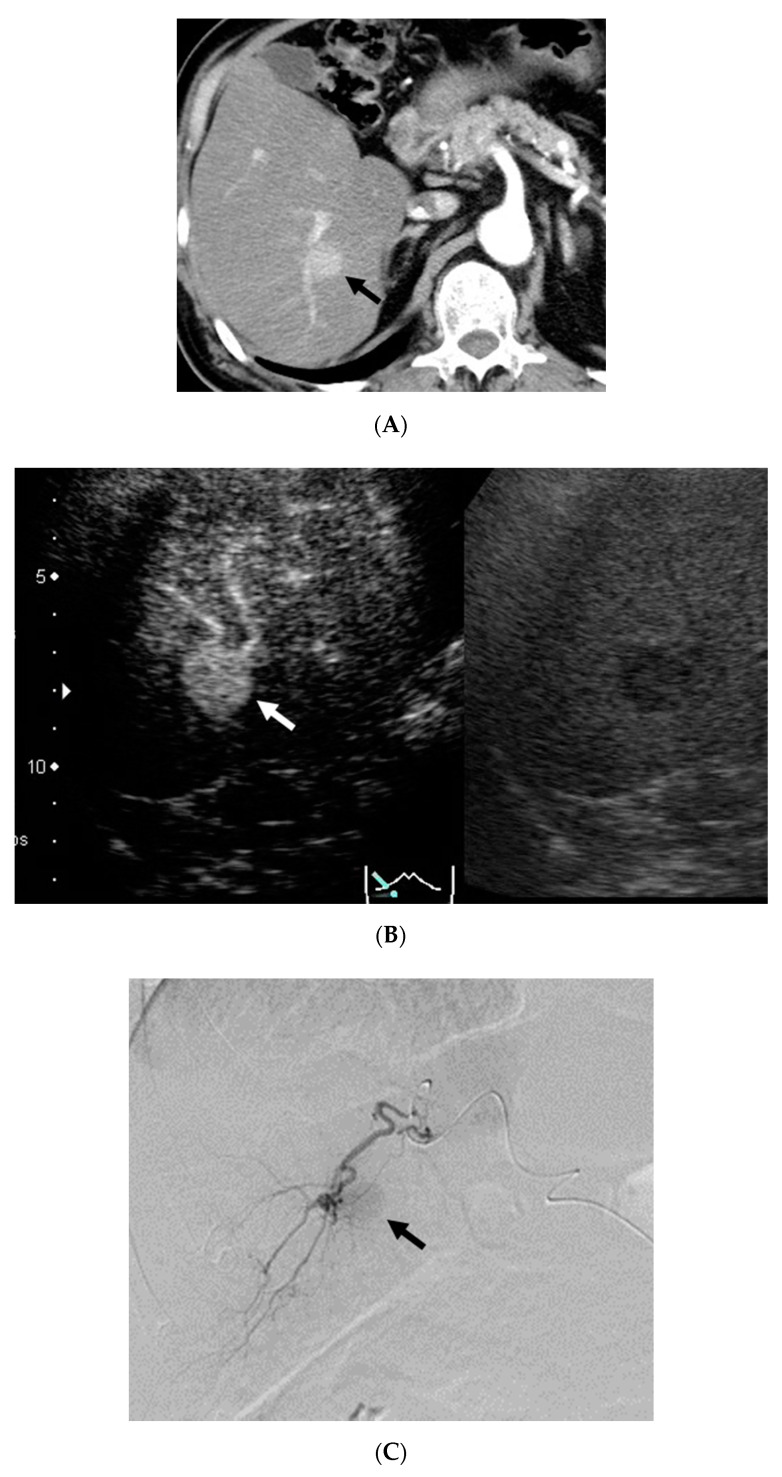

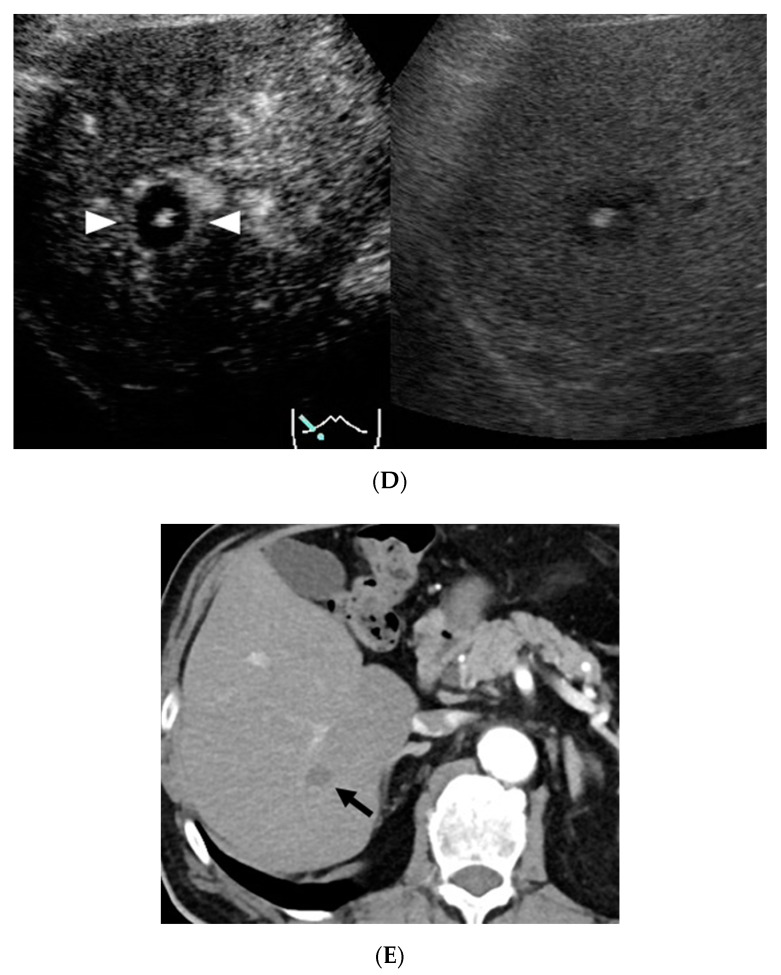
The patient was a 65-year-old male with alcoholic liver cirrhosis. DEB-TACE for HCC in S7 with a diameter of 20 mm and CEUS for the HCC within 3 days after DEB-TACE were performed. (**A**) Contrast-enhanced computed tomography (CECT) in the arterial phase before DEB-TACE showed a hypervascular lesion S7 (arrow). (**B**) CEUS in the vascular phase (40 s) before DEB-TACE showed a hyperenhanced lesion in S7 (arrow) (Right image: Monitor mode). (**C**) Digital subtraction angiography from a branch of A6 before DEB-TACE showed tumor stain (arrow). (**D**) CEUS in the vascular phase (40 s) showed peripheral ring enhancement (Pattern B) in S7 3 days after DEB-TACE (arrowhead). High echoic area in this lesion indicates that micro-air entered with beads during TACE (Right image: Monitor mode). (**E**) CECT in the arterial phase showed hypovascular lesion (complete response) in S7 12 months after DEB-TACE (arrow).

The enhancement patterns on CEUS performed within 3 days after DEB-TACE were defined as Pattern A in 17 cases, Pattern B in 7 cases (Figure 2), Pattern C in 13 cases, and Pattern D in 2 cases. Treatment efficacy assessed by CECT one month after DEB-TACE based on these patterns were A: CR16/PR1/SD0/PD0, B: CR6/PR1/SD0/PD0, C: CR2/PR6/SD3/PD2, and D: CR1/PR0/SD1/PD0. The CR rates at one month after treatment were 94.1% (16/17 lesions) for Pattern A, 85.7% (6/7) for Pattern B, 15.4% (2/13) for Pattern C, and 50% (1/2) for Pattern D. The 1-year local recurrence control rate was 53.0% in all 39 lesions.

The response rates were significantly higher for lesions with Pattern A compared to those with Pattern C at one month (17/17 vs. 8/13, *p* = 0.009) and 12 months (13/17 vs. 1/13, *p* < 0.001) after treatment, and significantly higher for lesions with Pattern B compared to those with Pattern C at 12 months after treatment (4/7 vs. 1/13, *p* = 0.031). Comparisons between other patterns showed no significant differences.

The median tumor sizes were 17 (8–35) for Pattern A, 26 (14–38) for B, 21 (15–50) for C, and (12–15) mm for D (*p* = 0.103). The median serum AFP levels were 6.1 (1.7–940.2) for Pattern A, 7.1 (3.3–298.8) for B, 9.4 (2.8–3887) for C, and (3.8–5.1) ng/mL for D (*p* = 0.856). The median serum DCP levels were 23 (15–360) for Pattern A, 52 (14–287) for B, 20.5 (15–842) for C, and (11–14) mAU/mL for D (*p* = 0.07). Comparisons between each pattern showed no significant differences.

The kappa value showed excellent agreement between the two reviewers (kappa value = 0.89).

## 4. Discussion

CT and MRI are commonly used during follow-up after TACE in patients with HCC [18,19]. However, it is difficult to assess the therapeutic efficacy of DEB-TACE by CT based on Lipiodol^®^ (Laboratoire Guerbet, Aulnay-Sous-Bois, France) (Lip) accumulation, unlike after Lip-TACE. Previously, we showed that DEB-TACE for HCC achieved a high CR rate [16]. Therefore, in order to accurately detect the feeding blood vessels, we performed TACE using IAUS—not only DEB-TACE but also conventional TACE. In addition, the possibility of sustained release of the chemotherapeutic agent also makes it difficult to predict the therapeutic efficacy of DEB-TACE. In particular, regions after DEB-TACE for HCC have different enhancements, and not all enhancements show viable lesions [8]. However, patients treated with TACE are likely to be in an advanced stage of disease and to need additional treatment without delay if therapeutic efficacy is insufficient. Therefore, early assessment after treatment is important to predict the therapeutic efficacy of DEB-TACE.

In this study, we performed CEUS with Sonazoid^®^ within 3 days after DEB-TACE for 39 HCC lesions and investigated whether enhancement patterns can be used to predict the early therapeutic efficacy of DEB-TACE. High CR rates at one month after treatment were found for lesions with no enhancement (Pattern A, 94.1%) and peripheral ring enhancement (Pattern B, 85.7%), and the CR rates at both 1 and 12 months after treatment were also significantly higher for lesions with no enhancement than for those with partial enhancement (Pattern C).

Shaw et al. [11] found that assessment of residual blood flow at 1–2 weeks and 1 month after treatment with DEB-TACE in 16 patients with HCC using CEUS with Definity^®^ (Lantheus Medical Imaging, North Billerica, MA, USA) was comparable to that of CT and MRI, and that CEUS was particularly useful for the assessment of early residual blood flow 1–2 weeks after treatment. Similarly, in the present study, CEUS was found to be effective for the assessment of early therapeutic efficacy within 3 days after DEB-TACE. Chung et al. [10] classified the enhancement in CECT during the arterial phase within one month after DEB-TACE in patients with HCC into three patterns: no enhancement, peripheral ring enhancement, and peripheral nodule-like enhancement. Cases with peripheral nodule-like enhancement had significant tumor progression, showing that pattern assessment by CECT in the arterial phase after TACE is useful to predict the treatment outcome. Similarly to the present study, these results suggest that therapeutic efficacy may be good if tumor enhancement completely disappears in imaging after treatment with DEB-TACE.

It is unclear if peripheral ring enhancement shows the presence of a residual tumor, since it may also reflect fibroinflammatory changes or pseudocapsular enhancement [10]. Residual tumors identified by peripheral ring enhancement may occur inside or outside treated regions [20,21], with enhancements inside treated regions in the arterial phase probably showing residual lesions [22]. Furthermore, uniform ring enhancement differs from non-uniform enhancement in assessment (i.e., uniform ring enhancement is found outside treated regions if the treatment was successfully completed) [10]. In this study, Pattern B was defined as complete ring enhancement, including a thin ring width and a location inside the treated tumor margin. A comparison between cases with Patterns B and C at 12 months post-treatment showed a significantly higher CR rate in those with Pattern B. Similarly to previous studies [10], it was observed that thin and uniform enhancement outside treated regions may reflect treatment-induced inflammation, rather than residual tumors.

In DEB-TACE in patients with HCC, no enhancement or peripheral ring enhancement in CEUS immediately after treatment may suggest good therapeutic efficacy, whereas other enhancement patterns may be predictors of recurrence that indicate that careful follow-up is needed in these cases.

These conclusions are limited by the small number of subjects, the short observation period, and the absence of histopathological analysis of peripheral ring enhancement. We intend to increase the number of subjects and extend the observation period to establish the value of the early assessment of therapeutic efficacy of DEB-TACE using enhancement patterns in CEUS.

## 5. Conclusions

CEUS immediately after DEB-TACE may allow for the early assessment of therapeutic efficacy, with findings of no enhancement or peripheral ring enhancement suggesting a positive outcome. Further studies in more subjects are required to validate these results.

## Figures and Tables

**Table 1:** Baseline patient and tumor characteristics. HBV: hepatitis B virus; HCV; hepatitis C virus; NASH: non-alcoholic steatohepatitis.

Characteristics	All (*n* = 32)
Age (years)(median)	72 (range 44–89)
Gender Male/female	28/4
Etiology	
Alcohol/HBV/HCV/NASH	14/4/12/2
Child-Pugh classification A/B	23/9
Previous treatment y/n	18/14
Tumor number	39
Tumor size(mm) (median)	21 (range 8–50)
DC Bead (100–300 μm, mL) (median)	0.6 (range 0.2–1.5)

## References

[1] Kakeda S., Korogi Y., Ohnari N., Moriya J., Oda N., Nishino K., Miyamoto W. (2007). Usefulness of cone-beam volume CT with flat panel detectors in conjunction with catheter angiography for transcatheter arterial embolization. J. Vasc. Interv. Radiol..

[2] Shiozawa K., Watanabe M., Ikehara T., Shimizu R., Shinohara M., Igarashi Y., Sumino Y. (2017). Evaluation of sorafenib for advanced hepatocellular carcinoma with low α-fetoprotein in arrival time parametric imaging using contrast-enhanced ultrasonography. J. Med. Ultrason..

[3] Shiozawa K., Watanabe M., Ikehara T., Kobayashi K., Ochi Y., Suzuki Y., Fuchinoue K., Yoneda M., Kenmochi T., Okubo Y. (2016). Evaluation of contrast-enhanced ultrasonography for hepatocellular carcinoma prior to and following stereotactic body radiation therapy using the CyberKnife® system: A preliminary report. Oncol. Lett..

[4] Llovet J.M., Bruix J. (2003). Systematic review of randomized trials for unresectable hepatocellular carcinoma: Chemoembolization improves survival. Hepatology.

[5] Lewis A.L., Gonzalez M.V., Leppard S.W., Brown J.E., Stratford P.W., Phillips G.J., Lloyd A.W. (2007). Doxorubicin eluting beads-1: Effects of drug loading on bead characteristics and drug distribution. J. Mater. Sci. Mater. Med..

[6] Maluccio M., Covey A. (2012). Recent progress in understanding, diagnosing, and treating hepatocellular carcinoma. CA Cancer J. Clin..

[7] Cazejust J., Bessoud B., Colignon N., Garcia-Alba C., Planché O., Menu Y. (2014). Hepatocellular carcinoma vascularization: From the most common to the lesser known arteries. Diagn. Interv. Imaging.

[8] Song M.J., Park C.H., Kim J.D., Kim H.Y., Bae S.H., Choi J.Y., Yoon S.K., Chun H.J., Choi B.G., Lee H.G. (2011). Drug-eluting bead loaded with doxorubicin versus conventional Lipiodol-based transarterial chemoembolization in the treatment of hepatocellular carcinoma: A case-control study of Asian patients. Eur. J. Gastroenterol. Hepatol..

[9] Kalva S.P., Iqbal S.I., Yeddula K., Blaszkowsky L.S., Akbar A., Wicky S., Zhu A.X. (2011). Transarterial chemoemboliza tion with Doxorubicin-eluting microspheres for inoperable hepatocellular carcinoma. Gastrointest. Cancer Res..

[10] Chung W.S., Lee K.H., Park M.S., Lee Y.J., Kwon J., Baek S.E., Kim M.J. (2012). Enhancement patterns of hepatocellular carcinoma after transarterial chemoembolization using drug-eluting beads on arterial phase CT images: A pilot retrospective study. Am. J. Roentgenol..

[11] Shaw C.M., Eisenbrey J.R., Lyshchik A., O’Kane P.L., Merton D.A., Machado P., Pino L., Brown D.B., Forsberg F. (2015). Contrast-enhanced ultrasound evaluation of residual blood flow to hepatocellular carcinoma after treatment with transarterial chemoembolization using drug-eluting beads: A prospective study. J. Ultrasound Med..

[12] Bruix J., Sherman M., American Association for the Study of Liver Diseases (2011). Management of hepatocellular carci noma: An update. Hepatology.

[13] Kokudo N., Hasegawa K., Akahane M., Igaki H., Izumi N., Ichida T., Uemoto S., Kaneko S., Kawasaki S., Ku Y. (2015). Evidence-based Clinical Practice Guidelines for Hepatocellular Carcinoma: The Japan Society of Hepatology 2013 update (3rd JSH-HCC Guidelines). Hepatol. Res..

[14] Oken M.M., Creech R.H., Tormey D.C., Horton J., Davis T.E., McFadden E.T., Carbone P.P. (1982). Toxicity and response criteria of the Eastern Cooperative Oncology Group. Am. J. Clin. Oncol..

[15] Kudo M., Hatanaka K., Maekawa K. (2010). Newly developed novel ultrasound technique, defect reperfusion ultrasound imaging, using sonazoid in the management of hepatocellular carcinoma. Oncology.

[16] Shiozawa K., Watanabe M., Ikehara T., Yamamoto S., Matsui T., Saigusa Y., Igarashi Y., Maetani I. (2018). Efficacy of intra-arterial contrast-enhanced ultrasonography during transarterial chemoembolization with drug-eluting beads for hepatocellular carcinoma. World J. Hepatol..

[17] Lencioni R., Llovet J.M. (2010). Modified RECIST (mRECIST) assessment for hepatocellular carcinoma. Semin. Liver Dis..

[18] Hammerstingl R., Huppertz A., Breuer J., Balzer T., Blakeborough A., Carter R., Fusté L.C., Heinz-Peer G., Judmaier W., Laniado M. (2008). Diagnostic efficacy of gadoxetic acid (Primovist)-enhanced MRI and spiral CT for a therapeutic strategy: Comparison with intraoperative and histopathologic findings in focal liver lesions. Eur. Radiol..

[19] Akai H., Kiryu S., Matsuda I., Satou J., Takao H., Tajima T., Watanabe Y., Imamura H., Kokudo N., Akahane M. (2011). Detection of hepatocellular carcinoma by Gd-EOB-DTPA-enhanced liver MRI: Comparison with triple phase 64 detector row helical CT. Eur. J. Radiol..

[20] Mannelli L., Kim S., Hajdu C.H., Babb J.S., Clark T.W., Taouli B. (2009). Assessment of tumor necrosis of hepatocellular carcinoma after chemoembolization: Diffusion-weighted and contrast-enhanced MRI with histopathologic correlation of the explanted liver. Am. J. Roentgenol..

[21] Kim S., Mannelli L., Hajdu C.H., Babb J.S., Clark T.W., Hecht E.M., Taouli B. (2010). Hepatocellular carcinoma: Assessment of response to transarterial chemoembolization with image subtraction. J. Magn. Reson. Imaging.

[22] Goldberg S.N., Grassi C.J., Cardella J.F., Charboneau J.W., Dodd G.D., Dupuy D.E., Gervais D., Gillams A.R., Kane R.A., Lee F.T. (2005). Image-guided tumor ablation: Standardization of terminology and reporting criteria. Radiology.

